# YTHDF2 suppresses the 2C-like state in mouse embryonic stem cells *via* the DUX-ZSCAN4 molecular circuit

**DOI:** 10.1016/j.jbc.2025.108479

**Published:** 2025-04-04

**Authors:** Xiang Wu, Wanting Cai, Junjie He, Shiyin Zhang, Shen Wang, Lingci Huang, Haotian Zhang, Xiaoyan Sun, Jun Zhou, Xiao-Min Liu

**Affiliations:** 1 School of Life Science and Technology, China Pharmaceutical University, Nanjing, Jiangsu, China; 2 Jiangsu Key Laboratory of Drug Design and Optimization, China Pharmaceutical University, Nanjing, China

**Keywords:** embryonic stem cell, YTHDF2, 2-cell like, m^6^A modification, RNA decay

## Abstract

Mouse embryonic stem cells (ESCs) consist of a rare population of heterogeneous 2-cell-like cells (2CLCs). These cells transiently recapitulate the transcriptional and epigenetic features of the 2-cell embryos, serving as a unique model for studying totipotency acquisition and embryonic development. Accumulating evidence has demonstrated that transcription factors and epigenetic modifications exert crucial functions in the transition of ESCs to 2CLCs. However, the roles of RNA modification in the regulation of the 2C-like state remain elusive. Using a DUX-induced 2CLCs system, we examine N^6^-methyladenosine (m^6^A) modification landscape transcriptome-wide and observe dynamic regulation of m^6^A during DUX-driven 2C-like reprogramming. Notably, many core 2C transcripts like *Dux* and *Zscan4* are highly methylated. We identify the m^6^A reader protein YTHDF2 as a critical regulator of 2C-like state. Depletion of YTHDF2 facilitates robust expression of 2C-signature genes and ESCs-to-2CLCs transition. Intriguingly, YTHDF2 binds to a subset of m^6^A-modified 2C transcripts and promotes their decay. We further demonstrate that YTHDF2 suppresses the 2C-like program in a manner that is dependent on both m^6^A and the DUX-ZSCAN4 molecular circuit. Mechanistically, YTHDF2 interacts with CNOT1, a key component of the RNA deadenylase complex. Consistently, silencing of CNOT1 upregulates the 2C program and promotes ESCs-to-2CLCs transition. Collectively, our findings reveal novel insights into the epitranscriptomic regulation of the 2C-like state in mouse ESCs.

Mouse embryonic stem cells (ESCs), also known as pluripotent stem cells, are derived from the inner cell mass of early blastocysts and possess the remarkable ability to differentiate into all embryonic tissues except extra-embryonic tissues (1, 2). Within the serum-cultured ESCs, there exists a certain degree of heterogeneity, which accounts for approximately 1% of the total cell population (3). These cells, designated as 2-cell-like cells (2CLCs), exhibit molecular signatures of transient totipotency. They demonstrate transcriptional activation of zygotic genome activation (ZGA) markers including the *Zscan4* cluster, *Dux*, and *MERVL* retrotransposons—molecular hallmarks classically associated with murine 2-cell embryos (4, 5, 6, 7). Functional analyses reveal that 2CLCs recapitulate key features of totipotent blastomeres, including chromatin accessibility remodeling and developmental potential spanning both embryonic and extraembryonic lineages (8, 9, 10). The bidirectional interconversion between pluripotent ESCs and totipotent-like 2CLCs establishes a valuable model system for studying the regulatory networks governing cell fate transitions during early mammalian development. Thus far, a multitude of regulatory factors have been identified that play significant roles in either promoting or inhibiting the transition from ESCs to the 2C-like cells. These factors include ZGA activation factors such as DPPA2/4 and NELFA (11, 12, 13), as well as epigenetic modifiers like CAF-1, SETDB1, and SMCHD1 (8, 14, 15), which participate in the regulation of transcriptional activity and epigenetic modifications, crucial for the transition from the pluripotent state to 2C-like state in mouse ESCs.

N^6^-methyladenosine (m^6^A) is the most prevalent modification within the eukaryotic mRNA (16). Through regulating various aspects of RNA metabolisms, such as degradation and translation (17, 18, 19), m^6^A plays crucial roles in physiological processes including embryonic development and cellular differentiation (20, 21, 22, 23). Recently, several studies have contributed to a more detailed depiction of m^6^A landscape during early mouse embryonic development, particularly highlighting the dynamic m^6^A alteration during the maternal-to-zygotic transition (24, 25, 26). In zebrafish, YTHDF2 specifically binds to certain m^6^A-modified maternal transcripts and promotes their degradation and its absence causes developmental defects (27). At present, few studies have focused on the roles of m^6^A in the regulation of 2C-like states in ESCs. As the unique nuclear m^6^A YTH reader protein, YTHDC1 suppresses the DUX-dependent 2C-like state through binding to m^6^A-modified transcripts of retrotransposons and subsequent chromatin modulation. As a result, knockout of *Ythdc1* or the m^6^A writer gene *Mettl3* induces the expression of 2C genes and 2C-like state transition (22, 28, 29). A very recent study suggests that the m^6^A-binding protein IGF2BP2 may control ESCs-to-2CLCs transition, as inhibition of IGF2BP2 by small molecule CW1-2 results in increased expression of 2C-related transcripts and expanded population of 2CLCs (30). However, the functional roles of cytoplasmic YTH readers in the regulation of 2C-like fate decisions remain elusive.

Here, we reveal the dynamic m^6^A landscape during DUX-induced reprogramming of ESCs into a 2C-like state by taking advantage of m^6^A sequencing. Notably, we identify the m^6^A reader protein YTHDF2 as a crucial suppressor of the 2C-like state. Depletion of YTHDF2 induces the activation of the 2C transcription program *via* both m^6^A and the DUX-ZSCAN4 regulatory circuitry. We further reveal that CNOT1 is potentially involved in YTHDF2-mediated stability of core 2C transcripts. Our findings highlight the importance of m^6^A in the regulation of the 2C-like reprogramming, providing crucial insights into epitranscriptomic modulation of the totipotent-like fate decision in mouse ESCs.

## Results

### Dynamic m^6^A profiles during 2C-like reprogramming

**Figure 1:**
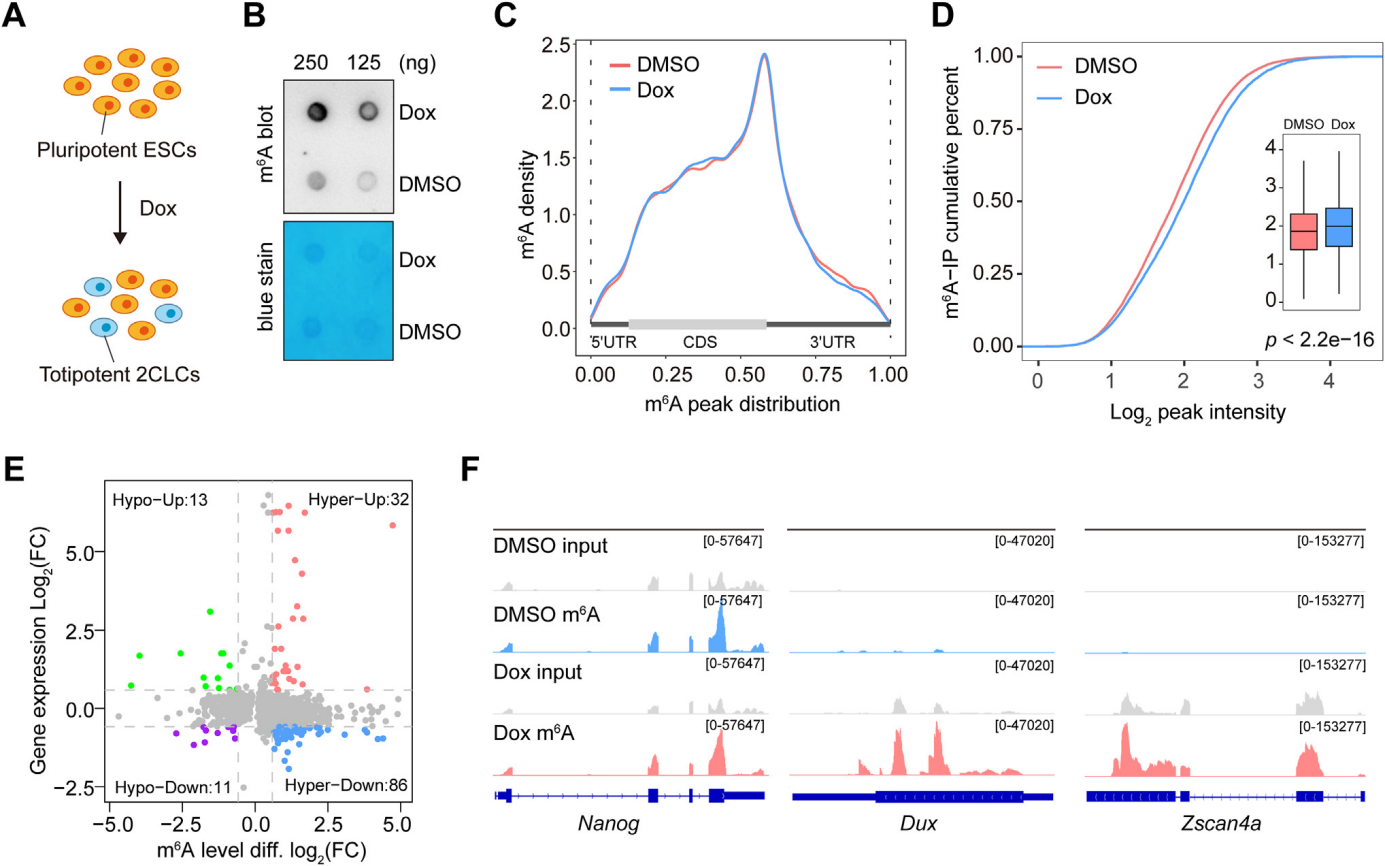
**Dynamic profiles of m^6^A modification during 2C-like reprogramming.***A*, a schematic representation of the inducible 2C-tdTomato system. Dox treatment induces the expression of exogenous *Dux*, robustly elevating the population of 2C-tdTomato-positive cells (2CLCs). *B*, dot blot analysis of m^6^A levels in ESCs treated with Dox or DMSO. Methylene blue staining is used as the loading control. *C*, the m^6^A distribution across the transcriptome of cells treated with Dox or DMSO. *D*, the cumulative distribution showing m^6^A peak intensity of cells treated with Dox or DMSO. *E*, four-quadrant chart analysis showing the distribution of transcripts with a significant change in both m^6^A levels and expression after Dox treatment (fold change > 1.5, *p* < 0.05). *F*, genome browser view of representative regions showing m^6^A peak density of *Nanog*, *Dux*, and *Zscan4a* in cells treated with Dox or DMSO.

RNA m^6^A modification plays crucial roles in a variety of biological processes. To elucidate the potential crosstalk between m^6^A and 2C-like reprogramming, we utilized the stable 2C-tdTomato transgenic mouse ESCs, which harbor both doxycycline (Dox)-inducible *Dux* transgene and *Mervl* promoter-driven tdTomato transgene (31). In this system, Dox treatment induced the expression of *Dux* and 2C-stage specific genes, facilitating the robust transition of ESCs to 2CLCs (Figs. 1*A* and S1). We initially examined the methylation state using m^6^A dot blotting. Unexpectedly, Dox treatment appeared to trigger an upregulation of RNA m^6^A levels (Fig. 1*B*). To systemically evaluate the global methylome during Dux-driven 2C-like reprogramming, we performed methylated RNA immunoprecipitation sequencing (m^6^A-seq) using cells treated with either DMSO or Dox. Consistent with previous studies, m^6^A sites were predominantly located in CDS and 3′UTR, with a notable enrichment near the stop codon (Figs. 1*C* and S2*A*). The canonical m^6^A motif, DRACH (D = A, G, or U; R = A or G; H = A, C, or U) was enriched in m^6^A peaks under both treatment conditions (Fig. S2*B*). In total, we identified 17,134 m^6^A peaks within 6068 transcripts for the DMSO group, and 12,002 m^6^A peaks within 4867 transcripts for the Dox group (Fig. S2, *C* and *D*). The m^6^A targets in DMSO and Dox groups were mainly enriched in the mRNA processing and ribonucleoprotein complex biogenesis pathways (Fig. S2*E*). Intriguingly, Dox-treated cells shared a substantial portion of methylated transcripts with 2-cell embryos (Fig. S2*F*). Remarkably, different m^6^A peak analyses revealed a total of 2639 differential m^6^A peaks distributed across 2060 genes, of which 2188 peaks distributed across 1688 transcripts were significantly upregulated (Fig. S2*G*), and 451 peaks distributed across 372 transcripts were substantially downregulated (Fig. S2*H*).

We then closely examined the m^6^A dynamics during 2C-like reprogramming. Consistent with the dot blotting assay, an obvious increase in global m^6^A methylation levels was observed upon Dox treatment (Fig. 1*D*). Integrative analysis of m^6^A-seq and RNA-seq data revealed four distinct groups: 118 hyper-methylated transcripts that were significantly up-regulated (hyper-up; n = 32) or down-regulated (hyper-down; n = 86), as well as 24 hypo-methylated transcripts that were significantly up-regulated (hypo-up; n = 13) or down-regulated (hypo-down; n = 11) (Fig. 1*E*). Consistent with previous studies (21, 22), the pluripotent transcript *Nanog* exhibited evident m^6^A peaks particularly near stop codon. Notably, the 2C-specific transcripts such as *Dux*, and *Zscan4a* were also heavily methylated in the form of m^6^A (Fig. 1*F*). Collectively, these results indicate that 2C-like reprogramming signal induces a global reshaping of the cellular mRNA methylome, suggesting a potential role of m^6^A in the regulation of the 2C-like state in ESCs.

### Depletion of YTHDF2 promotes the 2C-like transition dependently of m^6^A

**Figure 2:**
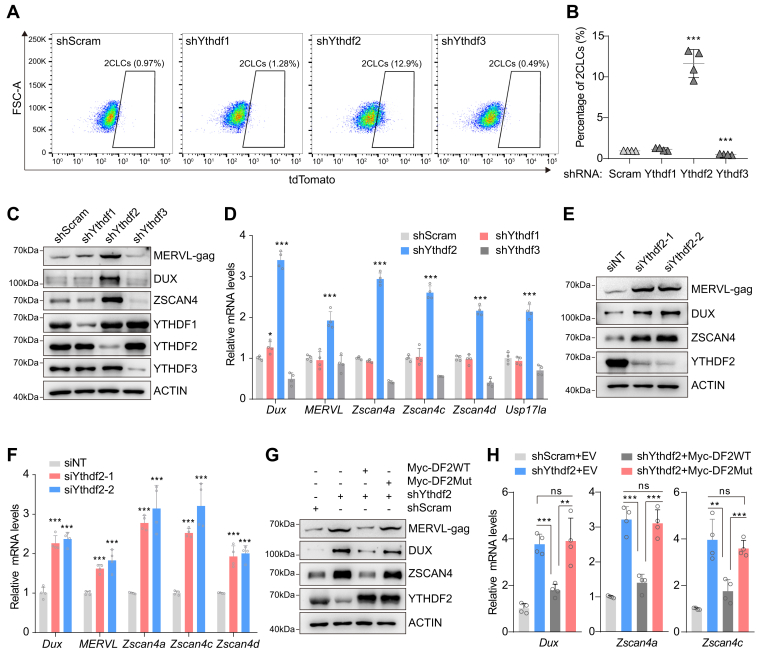
**Silencing of YTHDF2 promotes the 2C-like transition in a m^6^A-dependent manner**. *A*, Flow cytometry analysis showing the percentage of 2C-tdTomato-positive cells in *Ythdf*-KD and control ESCs. *B*, quantification of the percentage of 2C-tdTomato-positive cells in *Ythdf*-KD and control ESCs from four biologically independent experiments. *C*, Western blotting showing the expression of YTHDFs and 2C-related proteins in *Ythdf*-KD and control ESCs. *D*, detection of 2C gene expression by RT-qPCR in *Ythdf*-KD and control ESCs. *E*, Western blotting shows the expression of YTHDF2 and 2C-related proteins in cells transfected with siRNAs targeting *Ythdf2* or non-targeting siRNA (NT) as a control. *F*, detection of 2C gene expression by RT-qPCR in cells transfected with siRNAs targeting *Ythdf2* or non-targeting siRNA (NT) as a control. *G*, Western blotting showing the expression of YTHDF2 and 2C-related proteins in *Ythdf2*-KD cells transfected with genes encoding wild-type or m^6^A-binding mutant form of YTHDF2. *H*, detection of 2C gene expression by RT-qPCR in *Ythdf2*-KD cells transfected with genes encoding wild-type or m^6^A-binding mutant form of YTHDF2. Error bars, mean + SD; ∗*p* < 0.05, ∗∗*p* < 0.01, ∗∗∗*p* < 0.001, unpaired two tailed *t* test; *n* = 4 independent experiments.

m^6^A generally exerts cellular functions by recruiting its binding effectors. YTHDF family proteins have been identified as primary cytoplasmic m^6^A reader proteins (32). To explore the role of YTHDFs in the regulation of the 2C transcriptional program, we initially created stable *Ythdf*-deficient ESCs harboring *MERVL* promoter-driven tdTomato transgene using a lentiviral shRNA knockdown (KD) system. As shown in Figs. 2*C* and S3*A*, both mRNA and protein expression of *Ythdfs* were substantially decreased in *Ythdf-*KD cells. We next examined the 2C-like status of individual *Ythdf-*KD cells by flow cytometry. Compared to control cells, *Ythdf1*-KD and *Ythdf3*-KD cells showed negligibly altered and slightly reduced percentages of tdTomato-positive cells, respectively. Notably, the depletion of YTHDF2 resulted in a robust increase in the population of cells expressing 2C-tdTomato (Figs. 2, *A* and *B* and S3*B*). Consistent with the phenotype, *Ythdf2-*KD cells exhibited increased protein levels of MERVL-gag, DUX, and ZSCAN4, as well as significantly elevated expression of 2C-signature genes including *Dux*, *Zscan4a*, *Zscan4c*, *Usp17la*, and *MERVL*, compared to control cells (Fig. 2, *C* and *D*). Interestingly, the knockdown of *Ythdf2* also led to reduced gene expression of pluripotent factors (*Nanog*, *Sox2*, and *Pou5f1*) (Fig. S3*C*). To validate the effects of YTHDFs on the expression of 2C genes, we designed synthetic small interfering RNAs (siRNAs) to target individual *Ythdf* for interference. Again, the depletion of YTHDF2 rather than YTHDF1 or YTHDF3 resulted in robustly increased protein levels of MERVL-gag, DUX, and ZSCAN4 (Fig. S3*D*). We further independently confirmed the role of YTHDF2 in the regulation of 2C program by designing two distinct siRNAs targeting *Ythdf2*. Transfection of these siRNAs resulted in a dramatic reduction of YTHDF2 expression, and obviously increased mRNA and protein expression of *Dux*, *Zscan4,* and *MERVL* (Fig. 2, *E* and *F*). These results indicate that deficiency of YTHDF2 facilitates the 2C-like reprogramming in ESCs.

YTHDF proteins utilize YTH domains to recognize m^6^A marks and regulate target gene expression. To assess whether the m^6^A binding function of YTHDF2 was critical to the regulation of the 2C program, we rescued *Ythdf2-*KD cells with either wild-type or m^6^A binding mutant YTHDF2 constructs. Notably, the wild-type form rather than the mutant form rescued the increased expression levels of 2C-signature genes upon YTHDF2 depletion (Fig. 2, *G* and *H*). Taken together, these findings support the m^6^A-dependent role of YTHDF2 in the repression of the 2C-like state.

### Silencing of YTHDF2 upregulates the 2C program

**Figure 3:**
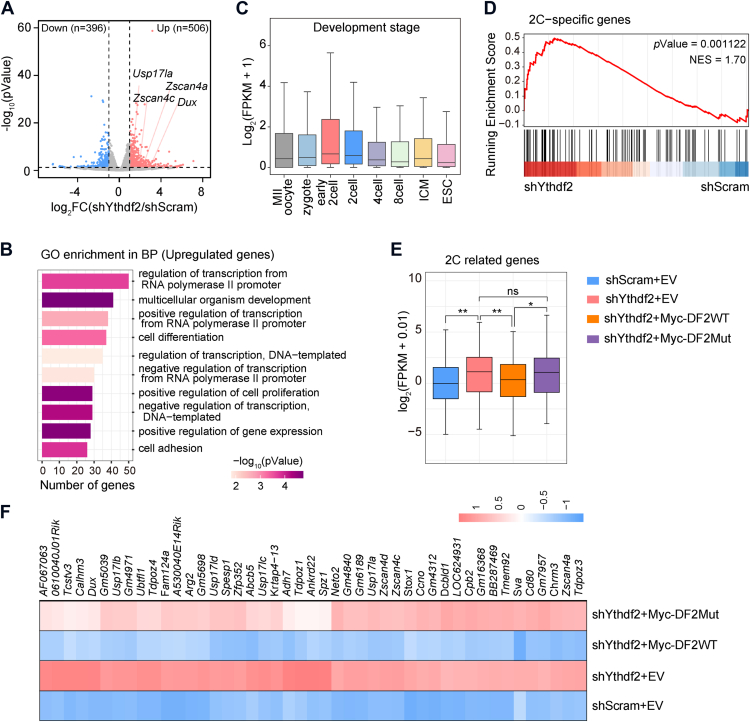
**Depletion of YTHDF2 upregulates the 2C-like transcriptome**. *A*, Volcano plots showing differentially expressed genes in response to YTHDF2 depletion. Upregulated genes (fold change > 2, *p* < 0.05) are marked in *red*, while downregulated genes (fold change < 0.5, *p* < 0.05) are marked in *blue*. *B*, GO analysis of the biological pathways enriched by upregulated genes upon YTHDF2 depletion. *C*, expression of upregulated genes in *Ythdf2*-KD ESCs at different preimplantation mouse embryo stages (GSE66582). *D*, GSEA using the 2-cell signature genes defined in (GSE33923) to compare *Ythdf2*-KD ESCs with control ESCs. *E*, Box plot showing the expression of 2C-related genes in *Ythdf2*-KD cells transfected with genes encoding wild-type or m^6^A-binding mutant form of YTHDF2. Significance (∗*p* < 0.05, ∗∗*p* < 0.01) was calculated with an unpaired two-tailed Student's *t* test. *F*, Heatmap showing the upregulation of many 2C-related genes in *Ythdf2*-KD cells rescued by wild-type rather than m^6^A-binding mutant form of YTHDF2.

To elucidate the role of YTHDF2 in the regulation of 2C-like reprogramming, we investigated the transcriptomic landscape in control and *Ythdf2-*KD ESCs. Two independent replicates showed a high Pearson correlation, indicating the reproducibility and reliability of RNA-seq datasets (Fig. S4*A*). In total, YTHDF2 knockdown induced significant downregulation of 396 genes and upregulation of 506 genes, including classic 2C-signature transcripts such as *Dux*, *Usp17la*, and *Zscan4* family genes (Fig. 3*A*). Notably, the increased expression of the MERVL family of endogenous retroviruses (MERVL-int and MT2_Mm) was also observed in *Ythdf2-*KD cells (Fig. S4*B*). GO analysis revealed that both significantly upregulated and downregulated genes were enriched in the pathways of cell differentiation and multicellular organism development (Figs. 3*B* and S4*C*). A close inspection of the upregulated genes revealed that they were highly expressed at 2-cell stages relative to all other developmental stages (Fig. 3*C*). In agreement with the role of YTHDF2 in the regulation of the 2C-like state in ESCs, 2C genes were significantly upregulated in *Ythdf2-*KD cells, revealed by gene set enrichment analysis (GSEA) (Fig. 3*D*). These observations suggest that YTHDF2 modulates the 2C program in transcriptome-wide.

We next investigate whether m^6^A binding activity is required for the YTHDF2-suppressed 2C program. The genes associated with the 2C program were identified and utilized based on previously published datasets (3, 29). Notably, we observed a significant upregulation of these 2C-related genes in *Ythdf2-*KD cells. Importantly, only the wild-type form of YTHDF2, but not its mutant variant, was able to efficiently rescue the elevated expression of these genes upon YTHDF2 depletion (Fig. 3, *E* and *F*). Collectively, these findings further suggest that YTHDF2 represses the 2C-like program in an m^6^A-dependent manner.

### YTHDF2 binds with core 2C transcripts and modulates their stability

**Figure 4:**
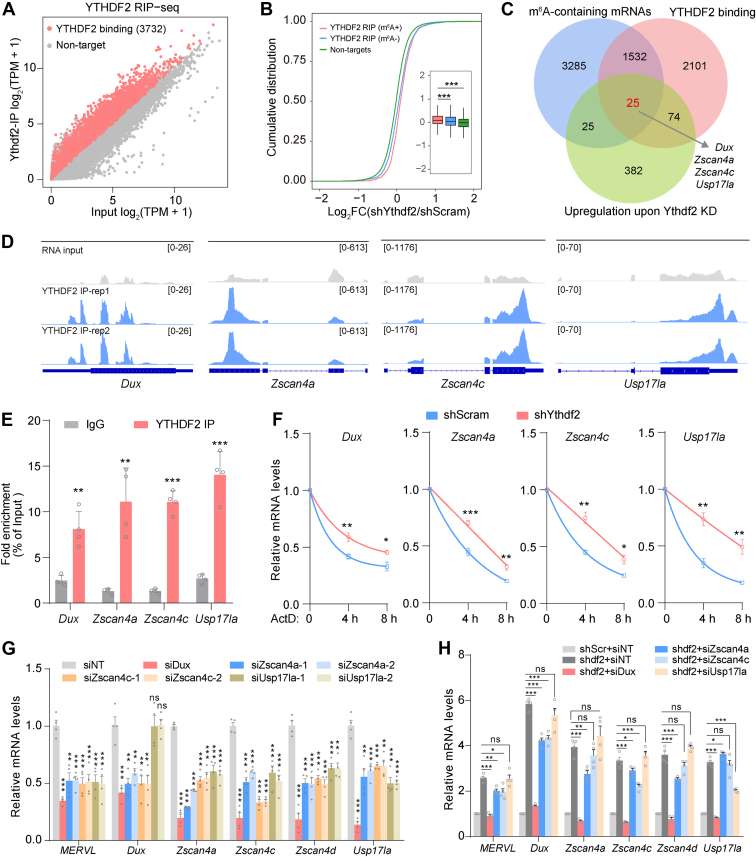
**YTHDF2 binds with key 2C transcripts and facilitates their decay**. *A*, scatter plot showing the normalized sequencing reads of RNA isolated from YTHDF2 IP or total RNA. *Red* dots represent enriched RNAs bound by YTHDF2 (fold change >2, *p* < 0.05), while the *grey* dots represent background RNAs. *B*, cumulative distribution of changes in transcript levels for YTHDF2 target mRNAs with m^6^A [YTHDF2 RIP (m^6^A+)], YTHDF2 target mRNAs without m^6^A [YTHDF2 RIP (m^6^A-)] and non-target mRNAs [Nontargets] between shScramble and shYthdf2. *C*, Venn diagram showing the overlap of transcripts identified by MeRIP-seq, YTHDF2-RIP-seq, and RNA-seq datasets. *D*, genome browser view of YTHDF2-binding transcripts *Dux*, *Zscan4a*, *Zscan4c*, and *Usp17la*. *E*, detection of YTHDF2-bound transcripts by YTHDF2 immunoprecipitation followed by RT-qPCR. *F*, detection of *Dux*, *Zscan4a*, *Zscan4c* and *Usp17la* transcript levels in *Ythdf2*-KD ESCs and control ESCs following treatment with the transcription inhibitor actinomycin D (ActD) for 0 h, 4 h, and 8 h. *G*, detection of the expression of indicated 2C genes by RT-qPCR in cells transfected with non-targeting siRNA (NT) or siRNAs targeting *Dux*, *Zscan4a*, *Zscan4c* and *Usp17la*. *H*, detection of the expression of indicated 2C genes by RT-qPCR in *Ythdf2*-KD cells transfected with non-targeting siRNA (NT) or siRNAs targeting *Dux*, *Zscan4a*, *Zscan4c* and *Usp17la*. The datasets were normalized relative to the control group (shScr + siNT). Error bars, mean + SD; ∗*p* < 0.05, ∗∗*p* < 0.01, ∗∗∗*p* < 0.001, unpaired two tailed *t* test; *n* = 4 independent experiments.

Acting as a m^6^A reader protein, YTHDF2 exerts cellular functions through direct recruitment of m^6^A-containing transcripts (17). The target transcripts of YTHDF2 have been identified under multiple cellular conditions, while its direct targets in the 2C program are yet to be determined. We therefore tended to capture the YTHDF2-binding targets in DUX-activated ESCs using RNA immunoprecipitation sequencing (RIP-seq). The results revealed that 3732 transcripts were pulled down by YTHDF2 (Fig. 4*A*). GO analysis revealed that these genes were enriched in the biological processes of transcription regulation and multicellular organism development (Fig. S5*A*). Using the Kyoto Encyclopedia of Genes and Genomes (KEGG) database, we found that the Hippo signaling pathway and signaling pathways regulating pluripotency of stem cells emerged as the primary enriched process for YTHDF2 target genes (Fig. S5*B*). As expected, transcripts bound by YTHDF2 exhibited greater mRNA expression compared with non-target mRNAs in response to YTHDF2 depletion (Fig. S5*C*).

Given the function of YTHDF2 in promoting the degradation of m^6^A-containing transcripts, we further cataloged the transcripts into three groups: non-targets, YTHDF2 RIP (m^6^A+), and YTHDF2 RIP (m^6^A-). Compared with non-targets, a noticeable increase of mRNA levels was observed in shYthdf2 cells for YTHDF2 targets both with and without m^6^A modifications. Intriguingly, among YTHDF2 targets, the transcripts with m^6^A (m^6^A+) exhibited more increased expression than those without m^6^A (m^6^A-) in response to YTHDF2 deficiency (Fig. 4*B*), further confirming the m^6^A-dependent role of YTHDF2 in mediating mRNA stability. To search for the potential direct targets of YTHDF2 in the regulation of 2C-like reprogramming, we performed the integrative analysis of the m^6^A-seq, RIP-seq, and RNA-seq datasets. In total, we identified an overlap of 25 transcripts, including 2C-specific genes such as *Dux*, *Zscan4a*/*c*, and *Usp17la* (Fig. 4*C*). Indeed, YTHDF2 binding peaks were significantly enriched in these transcripts (Fig. 4*D*). Independent validation by RIP-qPCR further revealed that YTHDF2 is directly bound with the *Dux*, *Zscan4a*/*c*, and *Usp17la* transcripts (Fig. 4*E*).

To elucidate the molecular mechanisms underlying how YTHDF2 regulates the expression of 2C transcripts, we initially confirmed the methylation status of *Dux*, *Zscan4a*/*c*, and *Usp17la* by m^6^A-RIP-qPCR. As shown in Fig. S5*D*, these transcripts were obviously methylated by m^6^A regardless of Dox treatment. To investigate whether YTHDF2 modulates the stability of *Dux*, *Zscan4a*/*c*, and *Usp17la* transcripts, we performed the mRNA decay assay after treatment with the transcription inhibitor actinomycin D (ActD). We found that the depletion of YTHDF2 significantly stabilized these 2C-specific transcripts (Fig. 4*F*). Collectively, the findings indicate that YTHDF2 binds m^6^A-modified *Dux*, *Zscan4a*/*c*, and *Usp17la* mRNAs to facilitate their decay, as a result, the absence of YTHDF2 increases their expression and subsequent 2C transcriptional program.

DUX and ZSCAN4C have been identified to activate 2C transcriptional program, facilitating 2C-like transition (7, 33). However, the functional roles of the other two YTHDF2 target genes, *Zscan4a* and *Usp17la*, in this process remain unclear. To examine the role of the above factors in the regulation of the 2C program, we designed siRNAs to interfere with the expression of individual genes. As expected, the silencing of DUX or ZSCAN4C resulted in a significant reduction in the expression levels of 2C genes, including *MERVL*, *Zscan4a*/*d, and Usp17la*. Similar to ZSCAN4C, knockdown of *Zscan4a* also downregulated the expression of *MERVL*, *Zscan4d*, and *Usp17la* (Fig. 4*G*). However, silencing of USP17LA led to an obvious decrease in the expression of *MERVL* and *Zscan4*, but not *Dux*, suggesting the existence of a positive feedback loop between DUX and ZSCAN4 in the regulation of 2C transcript expression, rather than involving USP17LA. To explore the interplay between *Dux*, *Zscan4*, and *Usp17la* in the regulation of the YTHDF2-suppressed 2C program, we silenced each gene in *Ythdf2-*KD ESCs. Depletion of DUX, other than USP17LA, fully reversed the upregulation of *MERVL*, *Zscan4a*, *Zscan4c*, and *Zscan4d* transcripts caused by YTHDF2 deficiency (Fig. 4*H*). While silencing of ZSCAN4A or ZSCAN4C partially complemented the upregulation of these genes caused by the depletion of YTHDF2. Altogether, these findings support a model in which YTHDF2 relies on both m^6^A and DUX-ZSCAN4 feedback circuits to repress the 2C-like program.

### Depletion of the YTHDF2-interacting factor CNOT1 drives the 2C-like state

**Figure 5:**
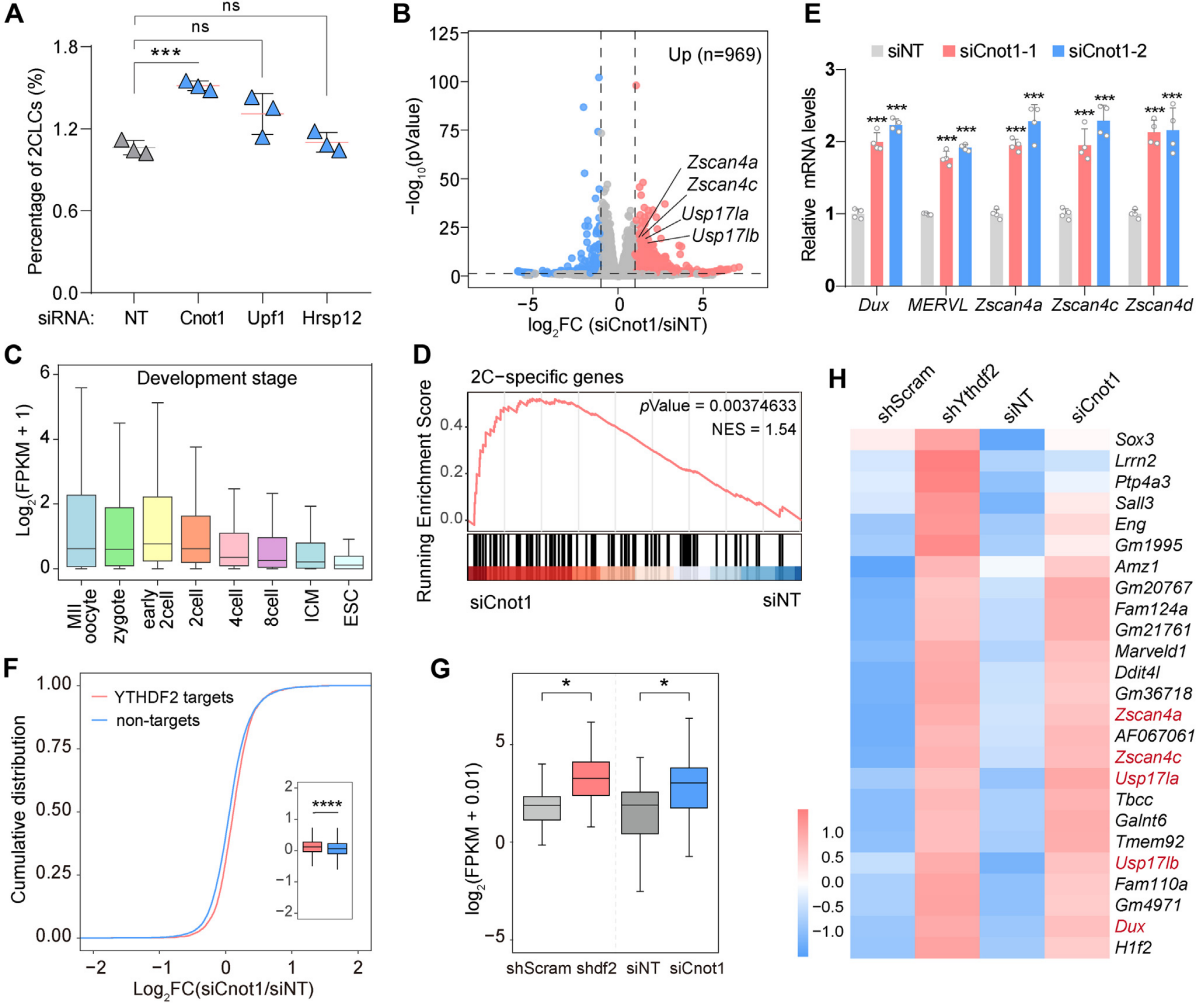
**YTHDF2-interacting factor CNOT1 represses the 2C-like program**. *A*, quantification of the percentage of 2C-tdTomato-positive cells in ESCs transfected with siRNA targeting *Cnot1*, *Upf1*, *Hrsp12* or non-targeting siRNA (NT) as a control from three biologically independent experiments. Error bars, mean ± SD; ∗∗∗*p* < 0.001, unpaired two-tailed *t* test. *B*, volcano plots showing differentially expressed genes in response to CNOT1 depletion. Upregulated genes (fold change > 2, *p* < 0.05) are marked in *red*, while downregulated genes (fold change < 0.5, *p* < 0.05) are marked in *blue*. *C*, expression of upregulated genes in *Cnot1*-KD ESCs at different preimplantation mouse embryo stages (GSE66582). *D*, GSEA using the 2-cell signature genes defined in (GSE33923) to compare *Cnot1*-KD ESCs with control ESCs. *E*, detection of 2C gene expression by RT-qPCR in ESCs transfected with siRNA targeting *Cnot1* or non-targeting siRNA. *F*, cumulative distribution of changes in transcript levels for YTHDF2 target mRNAs (YTHDF2 RIP) and non-target mRNAs (non-targets) between *Cnot1*-KD and control cells. *G*, box plot showing the expression of main YTHDF2-target transcripts (n = 25) in *Ythdf2*-KD, *Cnot1*-KD, and control cells. *H*, heatmap showing the consistent upregulation of YTHDF2-target transcripts (n = 25) in both *Ythdf2*-KD and *Cnot1*-KD cells. Error bars, mean + SD; ∗∗∗*p* < 0.001, unpaired two-tailed *t* test; *n* = 4 independent experiments.

The cytosolic m^6^A reader YTHDF2 has been shown to recruit regulatory factors to mediate mRNA degradation. In particular, YTHDF2 regulates the deadenylation and subsequent decay of m^6^A-containing RNAs through direct interaction with CNOT1, the major subunit of the CCR4-NOT complex (34). HRSP12 associates with YTHDF2 and RNase P/MRP to form a ternary complex, eliciting endoribonucleolytic cleavage of m^6^A-modified linear and circular RNAs (35). A recent study revealed that UPF1 interacts with YTHDF2 and triggers nonsense-mediated mRNA decay (36). To further gain insights into the regulatory mechanisms underlying YTHDF2-mediated stability of target 2C transcripts, we tended to investigate the effect of the above RNA decay factors on the 2C gene expression and 2C-like reprogramming. We designed a panel of siRNAs to individually target *Upf1*, *Hrsp12*, and *Cnot1* for expression interference. Of note, only the silencing of *Cnot1* led to in significant increase in the population of cells expressing 2C-tdTomato (Figs. 5*A* and S6*A*). Consistently, depletion of CNOT1 rather than UPF1 or HRSP12 resulted in dramatic upregulation of ZSCAN4 (Fig. S6*B*), suggesting that depletion of CNOT1 drives the 2C-like state in ESCs. We further confirmed the interaction between YTHDF2 and CNOT1 in ESCs. Indeed, endogenous YTHDF2 readily pulled down the CNOT1 subunit of the CCR4-NOT complex (Fig. S6*C*).

To systemically investigate the transcriptomic changes regulated by CNOT1, we performed RNA-seq for control and *Cnot1-*KD cells. In total, *Cnot1* knockdown induced significant upregulation of 969 genes, including 2C transcripts such as *Zscan4a*, *Zscan4c*, and *Usp17la* (Fig. 5*B*). Further analysis of these upregulated genes showed that they were enriched in the pathways of multicellular organism development, and expressed highly at the 2-cell stage compared to other developmental stages (Figs. 5*C* and S6*D*). Consistent with the role of CNOT1 in the negative regulation of 2C-like state, GSEA revealed that 2C genes were significantly upregulated in *Cnot1-*KD cells (Fig. 5*D*). We further independently confirmed the role of CNOT1 in the suppression of 2C gene expression by designing two distinct siRNAs. Transfection of both siRNAs consistently upregulated the expression of *MERVL*, *Dux*, and *Zscan4* genes, as well as increased the population of 2CLCs (Figs. 5*E* and S6, *E* and *F*). These data indicate that depletion of CNOT1 facilitates the expression of 2C-related transcripts and 2C-like transition. We next investigated the regulatory role of YTHDF2 and CNOT1 in the modulation of the 2C-like state. Upon depletion of CNOT1, YTHDF2-bound mRNAs exhibited elevated expression levels compared to non-target mRNAs (Fig. 5*F*), suggesting a cooperative mechanism between YTHDF2 and CNOT1 in regulating YTHDF2-targeted transcripts. We previously identified 25 2C-related genes as key targets for YTHDF2 (Fig. 4*C*). Notably, these genes showed consistent upregulation in *Ythdf2-*KD and *Cnot1-*KD cells (Fig. 5, *G* and *H*). Based on these observations, we conclude that YTHDF2 could collaborate with CNOT1 to suppress the expression of 2C-specific transcripts and the 2C-like state in ESCs.

## Discussion

2CLCs are a small fraction of heterogeneous cells found in mouse ESCs under culture conditions. They display a similar transcriptional program as the 2-cell blastomere, capable of generating both embryonic and extra-embryonic tissues (3, 37). DUX is a master transcription factor crucial for driving the expression of a multitude of 2C-specific genes and retrotransposons such as *Zscan4* and *MERVL*, and establishing the 2C-like state (7, 38). The DUX-based inducible 2CLCs model has been extensively used to examine the transcriptional dynamics of the 2C-like transition and identify crucial factors regulating totipotency and the cell state transition process (31, 39). A growing body of evidence has revealed that a set of transcriptional and epigenetic factors are involved in the regulation of 2C-like state in ESCs (9). However, the role of epitranscriptomic factors in this process is still poorly understood.

By taking advantage of the Dox-inducible system, we present the profile of RNA m^6^A dynamics during DUX-driven the transition of pluripotent ESCs to totipotent 2CLCs and reveal an increase of methylation levels transcriptome-wide (Fig. 1, *B* and *C*). Notably, several recent reports have drawn the dynamic landscapes of m^6^A RNA modifications during mouse oocyte maturation and early embryonic development. Particularly, they found that m^6^A exerts an important function in the regulation of maternal-to-zygotic transition (24, 25, 26), highlighting the critical roles of m^6^A-guided post-transcriptional regulation during early embryonic development. In our study, we uncover that a group of vital 2C-signature transcripts such as *Dux* and *Zscan4*, is subject to the modification by m^6^A (Fig. 1*F*). The methylated feature of *Dux* and *Zscan4* implies that m^6^A might have a role in the metabolic modulation of these target transcripts.

YTH domain-containing proteins are primary m^6^A readers that play important roles in the regulation of methylated RNA metabolism (40). While the role of nuclear reader YTHDC1 in the 2C-like program is well established (28, 29), how the cytoplasmic YTH reader in the regulation of this process is poorly understood. Our study identifies YTHDF2 as a critical regulator of the 2C-like state in ESCs. Specifically, silencing of YTHDF2 facilitates the expression of 2C-signature genes and the spontaneous transition of ESCs into the 2C-like state, thereby enhancing the population of 2CLCs (Fig. 2, *A*–*F*). Consistent with the phenotype, transcriptomic analysis reveals that YTHDF2 deficiency triggers the upregulation of a cluster of genes and endogenous retroviruses that are highly expressed at 2-cell stages, such as *Dux*, *Zscan4*, *Usp17la*, and *MERVL* (Figs. 3, *A* and *D* and S4*B*). YTHDF2 has been demonstrated to exert cellular functions through mediating metabolic changes of m^6^A-modified transcripts (32). We show that m^6^A-binding activity is required for YTHDF2-mediated regulation of the 2C-like state, as compared with the wild-type form, the mutation in the YTH domain of YTHDF2 fails to restore the abnormal expression of 2C genes caused by YTHDF2 depletion (Figs. 2, *G* and *H* and 3, *E* and *F*). These observations imply that YTHDF2-suppressed the transition of 2C-like state relies on m^6^A.

YTHDF2 is an RNA-binding protein that specifically recognizes and destabilizes m^6^A-modified transcripts. Integrative analysis of m^6^A-seq, YTHDF2 RIP-seq, and RNA-seq datasets reveals that the crucial 2C transcripts, *Dux*, *Zscan4*, and *Usp17la*, are not only modified by m^6^A, but also bound and regulated by YTHDF2 (Figs. 4, *A*–*E* and S5*D*). We next examine whether YTHDF2 regulates the stability of these target mRNAs. Intriguingly, the silencing of YTHDF2 dramatically retards the decay rate of *Dux*, *Zscan4*, and *Usp17la* transcripts (Fig. 4*F*). Collectively, these findings suggest that YTHDF2 may repress the 2C-like state by targeting and facilitating the degradation of these m^6^A-modified 2C transcripts. Notably, DUX, as a master transcription factor of the 2C program, is known to be transcriptionally regulated by various upstream factors (41, 42). Our study uncovers a potential role for YTHDF2 in regulating *Dux* expression through an m^6^A-dependent RNA degradation pathway, thereby revealing a novel post-transcriptional regulatory mechanism of DUX.

ZSCAN4 comprises a cluster of paralogs including ZSCAN4A-F and three pseudogenes. With robust expression at the 2-cell stage blastomere, ZSCAN4 has been shown to induce 2C gene expression and 2C-like transition through regulation of DNA demethylation and telomere elongation (4, 5, 43). Intriguingly, overexpression of ZSCAN4C has been found to upregulate the expression of *Dux*. Consistent with this, we found that ZSCAN4C or DUX could reciprocally regulate each other's expression as well as the expression of 2C genes, including *MERVL*, *Zscan4a*/*d, Usp17la* (Fig. 4*G*), indicating the presence of a positive feedback loop between ZSCAN4 and DUX in the regulation 2C-like program. Intriguingly, the knockdown of *Usp17la* resulted in a dramatic decrease in the expression of *MERVL* and *Zscan4* rather than *Dux*, suggesting a DUX-independent role of USP17LA in the regulation of 2C transcripts. Furthermore, we revealed that depletion of DUX or ZSCAN4, but not USP17LA, could fully or partially rescue the upregulation of 2C-signature transcripts caused by deficiency of YTHDF2, respectively (Fig. 4*H*). Collectively, these results indicate that depletion of YTHDF2 facilitates 2C gene expression and 2C-like state transition *via* dual mechanisms involving the selective stabilization of m^6^A-modified 2C transcripts and the DUX-ZSCAN4 feedback regulation. The DUX-ZSCAN4 molecular circuit exists to reinforce 2C-like reprogramming in *Ythdf2*-KD cells.

YTHDF2 has been documented to interact with diverse RNA decay machinery to mediate m^6^A-dependent mRNA turnover (34, 35, 36). However, our findings identify CNOT1, the scaffolding subunit of the CCR4-NOT deadenylase complex, as the primary functional partner of YTHDF2 in suppressing the 2C-like state. Previous studies have demonstrated that YTHDF2 directly binds CNOT1 through its N-terminal domain (34). Consistently, we also detected the interaction between YTHDF2 and CNOT1 in mouse ESCs. Importantly, the silencing of *Cnot1* led to enhanced expression of 2C-related genes including *MERVL*, *Dux*, and *Zscan4*, and promoted a 2C-like transition in ESCs (Fig. 5, *A*–*E*). Similarly, the knockdown of *Cnot10*, an auxiliary CCR4-NOT subunit, partially derepressed subsets of 2C-associated genes, such as *Zscan4c* and *Zscan4d* (44). These observations suggest that YTHDF2 and CCR4-NOT act cooperatively to destabilize 2C-specific mRNAs. Further supporting this mechanistic synergy, transcriptomic profiling revealed the consistent upregulation of primary YTHDF2-target transcripts in both *Ythdf2-*KD and *Cnot1-*KD cells (Fig. 5, *G* and *H*). Collectively, these results establish the CCR4-NOT complex as a crucial effector of YTHDF2 in restraining totipotency-associated programs.

**Figure 6:**
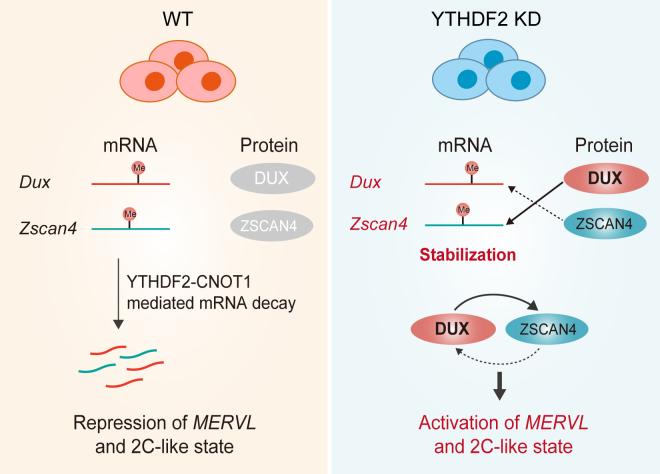
**Proposed working model of YTHDF2 in suppressing the 2C-like state.** A schematic model shows that YTHDF2 suppresses the 2C-like state in a manner that is dependent on both m^6^A and DUX-ZSCAN4 molecular circuits. In wild-type ESCs, YTHDF2 recruits the CCR4-NOT deadenylase complex (*via* CNOT1) to target m^6^A-modified 2C-related transcripts (*Dux* and *Zscan4*) for degradation. This process leads to the suppression of *MERVL* retroelements and the 2C-like state. Conversely, in the absence of YTHDF2, these 2C transcripts become stabilized and translate into functional proteins. The accumulated DUX and ZSCAN4 proteins then engage in a self-reinforcing molecular circuit, further amplifying the 2C-like reprogramming in YTHDF2-deficient cells.

In summary, our findings identify the negative role of YTHDF2 in the regulation of 2C-like fate decisions through both the m^6^A regulatory pathway and the DUX-ZSCAN4 circuit (Fig. 6). The functional interplay between YTHDF2 and m^6^A constitutes a vital component of the molecular machinery that modulates the 2C-like state. Our study highlights the significance of m^6^A in the regulation of 2C-like programs, providing deeper insights into the additional layers of totipotent-like regulations.

## Experimental procedures

### Cell culture

The ESCs containing tdTomato transgene driven by MERVL-promoter, kindly provided by Professor Xudong Fu of Zhejiang University. These cells were cultured on 0.2% gelatin-coated dishes in Knock-out DMEM (Gibco), 15% KnockOut Serum Replacement (Gibco), 1% non-essential amino acids (Gibco), 1% Glutamax (Gibco), 1% penicillin/streptomycin (Life Technologies), 0.1 mM β-mercaptoethanol (Invitrogen), and 1000 U/ml Recombinant Mouse leukemia inhibitory factor (LIF) Protein (Millipore). For ESC maintenance, the medium was replaced daily, and routine passaging was performed when cell confluency reached 70%.

### Generation of stable YTHDF knockdown mouse ESCs

The shRNA targeting sequences were cloned into pRSI9-U6-(sh)-UbiC-TagRFP-2A-Puro (Cellecta). Lentiviral particles were packaged using Lenti-X 293T cells. Virus-containing supernatants were collected at 48 h post-transfection and filtered to eliminate cell debris. Mouse ESCs were infected by the lentivirus for 48 h and stable ESC knockdown lines were generated using puromycin selection (0.5 μg/ml). shRNA targeting sequences are listed below:

shScramble: 5′-AACAGTCGCGTTTGCGACTGG-3′.

shYthdf1: 5′-GGACATTGGTACTTGGGATAA-3′

shYthdf2-1: 5′-GCAAACTTGCAGTTTATGTAT-3′

shYthdf2-2: 5′-GGACGTTCCCAATAGCCAACT-3′

shYthdf3: 5′-GGTGGGCTTCACCAATTAATG-3′

### siRNA transfection

The ESCs were seeded onto 35 mm culture dishes 4 h prior to reaching around 30% confluence at the time of transfection. All siRNA transfections were performed using LipofectamineTM 3000(Invitrogen, L3000015) according to the manufacturer’s instructions. Briefly, Lipofectamine 3000 reagent and siRNAs were separately diluted in Opti-MEM medium (Gibco, 11058021). Then, the diluted siRNAs were added to the diluted Lipofectamine 3000 reagent, followed by 15 min incubation at room temperature. Subsequently, the mixtures were added to the cell suspension at a final concentration of 150 nM siRNA. The cells were collected and analyzed 48 h after the first transfection.

siYthdf1: 5′-GCACUGACUGGUGUCCUUU(dT) (dT)-3′

siYthdf2-1: 5′-CCAUGCCCUAUCUAACUUCUU(dT) (dT)-3′

siYthdf2-2: 5′-GCAUGAAUACUAUAGACCA(dT) (dT)-3′

siYthdf3: 5′-GCAGUGGUAUGACUAGCAU(dT) (dT)-3'

siDux: 5′-GGAUCCUAGGGCAAGCCUU(dT) (dT)-3'

siZscan4a-1: 5′-GAGUUGAGGUGGAGGAAUA(dT) (dT)-3′

siZscan4a-2: 5′-CCUGAGUGCUCUGACUACU(dT) (dT)-3′

siZscan4c-1: 5′-CUGACAUUUUCUACAUGUU(dT) (dT)-3′

siZscan4c-2: 5′-ACGACCCAAGGGAGGUACC(dT) (dT)-3′

siUsp17la-1: 5′-GGAGCUAACUGUCAAUGGA(dT) (dT)-3′

siUsp17la-2: 5′-GGGAUAAGAGAGCAAUUAA(dT) (dT)-3′

siCnot1-1: 5′-GUUAGAGGCUUACGUUAAA(dT) (dT)-3′

siCnot1-2:5′-GACUACGUGCGAACAGAUA(dT) (dT)-3′

siUpf1: 5′-CGUCCAACAUCUUCUACGA(dT) (dT)-3′

siHrsp12: 5′-GCUUACCAAGUCGCUGCUUUA(dT) (dT)-3′

### Flow cytometry analysis

Twenty-four hours prior to experimentation, ESCs were seeded onto 35-mm culture dishes. The following day, cells were detached using 0.05% Trypsin-EDTA, centrifuged to remove the culture medium, washed once with 1 ml of 1 × PBS buffer, and resuspended in PBS buffer. Cells were then filtered through a 300-mesh sieve into a flow cytometry tube and immediately subjected to analysis using the FACSCelesta flow cytometer (Becton Dickins). Data and images were analyzed using FlowJo software (V10.8).

### RNA isolation and real-time quantitative PCR

Total RNA was isolated from cells using Trizol reagent (Invitrogen) according to the manufacturer's instructions. Isolated RNA was reverse transcribed into cDNA using the HiScript III first Strand cDNA Synthesis Kit plus gDNA wiper (Vazyme Biotech) to remove genomic DNA contamination. Real-time qPCR experiments were performed using ChamQ SYBR qPCR Master Mix (Vazyme Biotech) and qPCR detection was conducted using the QuantStudio three Real-Time PCR System (Applied Biosystems). Primers for RT-qPCR assay are listed in Table S1. All the qPCR data were normalized to *Actin* as an internal control using the 2^-ΔΔCt^ method.

### Western blot

For Western blot analysis, cells were lysed using 2× SDS loading sample buffer and boiled at 100°C for 10 min to denature the proteins. Subsequently, proteins were separated by SDS-PAGE gel electrophoresis and transferred onto PVDF membranes. The membranes were then blocked with 0.5% skim milk in TBST for 1 h at room temperature. Following blocking, the membranes were incubated overnight at 4 °C with primary antibodies. The next day, after washing, the membranes were incubated with secondary antibodies for 1 h at room temperature. Immunoblot signals were visualized using an enhanced chemiluminescence system (TANON SCIENCE&TECHNOLOGY). Below are the antibodies utilized in the experiments: anti-YTHDF1 (Proteintech, 17479-1-AP), anti-YTHDF2 (Proteintech, 24744-1-AP), anti-YTHDF3 (Proteintech, 25537-1-AP), anti-ZSCAN4 (Abclonal, A10205), anti-MERVL-gag (Beyotime, AF0240), anti-DUX (HUABIO, ER1901–52), anti-CNOT1 (Proteintech, 14276-1-AP).

### m^6^A dot blot

Total RNA was extracted using Trizol reagent (Thermo Fisher Scientific), followed by mRNA isolation from the total RNA using oligo-dT beads (New England BioLabs, S1419S) to deplete rRNA. The mRNA was transferred onto a nylon membrane, and a gradient was set up with 500 ng, 250 ng, and 125 ng. Subsequently, the dried nylon membrane was placed in a UV cross-linker and fixed, with parameters set to 0.3 J and a wavelength of 256 nm. After sealing at room temperature with 5% skim milk on a shaker at 50 rpm for 1 h, m^6^A antibody (Abcam, ab151230) was applied and incubated overnight at 4 °C on the shaker at 50 rpm. The next day, the corresponding secondary antibody was applied and incubated on the shaker at 50 rpm for 1 h. Finally, imaging was conducted using a chemiluminescence imaging system.

### RNA decay assay

The cells from the shYthdf2 and shControl groups, both in a healthy growth state, were cultured until reaching 70% ∼ 80% confluency. Upon reaching this density, cells were digested using 0.05% Trypsin-EDTA and then seeded onto 35 mm culture dishes at similar cell numbers for both groups. The following day, fresh medium was replaced, supplemented with Actinomycin D (ActD) at a final concentration of 5 μg/ml. Cells were harvested at 0 h, 4 h, and 8 h after ActD treatment using Trizol, and an equal amount of Luc mRNA was added. The degradation rate of relevant mRNA was determined using qPCR.

### RNA-seq and data analysis

Total RNA extracted using Trizol is subjected to poly(A)+ RNA isolation for the preparation of RNA-seq libraries. Two independent biological replicates are prepared for each sample group. The constructed libraries are then subjected to deep sequencing using the Illumina Novaseq6000 platform. Using the DESeq2 package (1.40.2), genes were identified as significantly differentially expressed if they met the criteria of *p*value ≤ 0.05 and |log2foldchange| ≥ 1. The significantly differentially expressed genes were subjected to GO pathway enrichment and KEGG analysis using the DAVID website (https://david.ncifcrf.gov/). Gene Set Enrichment Analysis (GSEA) was performed on the 2C gene set using the enrichplot package (1.20.0). To analyze gene expression across various developmental stages, we first identified genes that were significantly upregulated in RNA-seq experiments following the knockdown of either *Ythdf2* or *Cnot1*. Subsequently, we examined the expression levels (FPKM) of these genes at different preimplantation developmental stages, utilizing gene expression profiles sourced from the GSE66582 dataset.

### MeRIP-seq and data analysis

Total RNA was extracted using Trizol reagent (Thermo Fisher Scientific) and mRNA was subsequently isolated from the total RNA using oligo dT beads (New England BioLabs, S1419S) to remove rRNA. The mRNA was then subjected to chemical fragmentation by incubating at 94 °C for 5 min in a fragmentation buffer (10 mM Tris-HCl, 10 mM ZnCl_2_, pH 7.0), yielding fragments of approximately 150 nucleotides. The reaction was terminated by adding 0.05 M EDTA, followed by standard ethanol precipitation to purify the fragmented RNA. Enrichment of m^6^A-modified mRNA was carried out using anti-m^6^A antibody (Abcam, ab151230). Input mRNA and m^6^A-modified RNAs were subjected to library construction using the TruSeq Stranded mRNA Library Preparation kit (Illumina), following the manufacturer's recommendations. Sequencing was performed on the Illumina Novaseq6000 platform (LC-Bio Technology Co, Ltd) with paired-end reads (PE150). Reads were filtered using fastp (0.19.4), and the filtered reads were aligned to the mouse genome (mm10) using hisat2 software (2.2.1). m^6^A peak calling was performed using exomePeak2 software to identify m^6^A peaks, followed by motif analysis of m^6^A peaks using HOMER software.

### RIP-seq and data analysis

ESCs were collected in lysis buffer (10 mM HEPES pH 7.4, 5 mM MgCl2, 100 mM KCl, 1 mM DTT, 1:100 protease inhibitor cocktail, 400 U/ml^–1^ RNase inhibitor) on ice for 20 min. The lysates were then centrifuged at 13,000 rpm for 10 min to remove insoluble precipitates. Anti-YTHDF2 was added to the cell lysates and incubated overnight at 4 °C. The following day, 15 μl each of protein A and protein G magnetic beads were added to the cell lysates and incubated with rotation at 4 °C for 2 h. Subsequently, the cell lysates were discarded on a magnetic rack, and the beads were washed 7 times with high salt wash buffer (10 mM HEPES pH 7.4, 5 mM MgCl2, 250 mM KCl, 1 mM DTT), each wash lasting for 5 min. RNA enriched by immunoprecipitation (IP) was extracted using Trizol and utilized for library construction. The constructed libraries were sequenced on the Illumina Novaseq6000 platform, and the sequenced reads were aligned to the mouse genome mm10 using hisat2 software. DESeq2 was employed to identify peaks associated with YTHDF2 binding.

## Data availability

The m^6^A-seq, RNA-seq and RIP-seq data generated for this publication have been deposited in the National Center for Biotechnology Information Gene Expression Omnibus (GEO) and are accessible through the GEO Series accession number GSE270884 (token password: srinuiegftkpbox).

## Supporting information

This article contains supporting information.

## Conflict of interest

The authors declare that they have no conflicts of interest with the contents of this article.
