# Supplementary material for: YTHDF2 suppresses the 2C-like state in mouse embryonic stem cells via the DUX-ZSCAN4 molecular circuit
Source: J Biol Chem. 2025 Apr 4;301(5):108479. doi: 10.1016/j.jbc.2025.108479 (PMC12147224; doi:10.1016/j.jbc.2025.108479)
Supplement: Supporting information [file mmc1.pdf]

Supporting information

YTHDF2 suppresses the 2C-like state in mouse embryonic stem cells *via* the DUX-ZSCAN4 molecular circuit

Xiang Wu[1†], Wanting Cai[1†], Junjie He[1†], Shiyin Zhang[1], Shen Wang[1], Lingci Huang[1], Haotian Zhang[1], Xiaoyan Sun[1], Jun Zhou[1,2*], Xiao-Min Liu[1,2*]

[1]School of Life Science and Technology, China Pharmaceutical University, Nanjing, Jiangsu 210009, China

[2]Jiangsu Key Laboratory of Drug Design and Optimization, China Pharmaceutical University, Nanjing 21009, China

[†]These authors have contributed equally to this work.

[*]Correspondence: jz572@cpu.edu.cn (J.Z.), liuxm642@cpu.edu.cn (X.-M.L.)

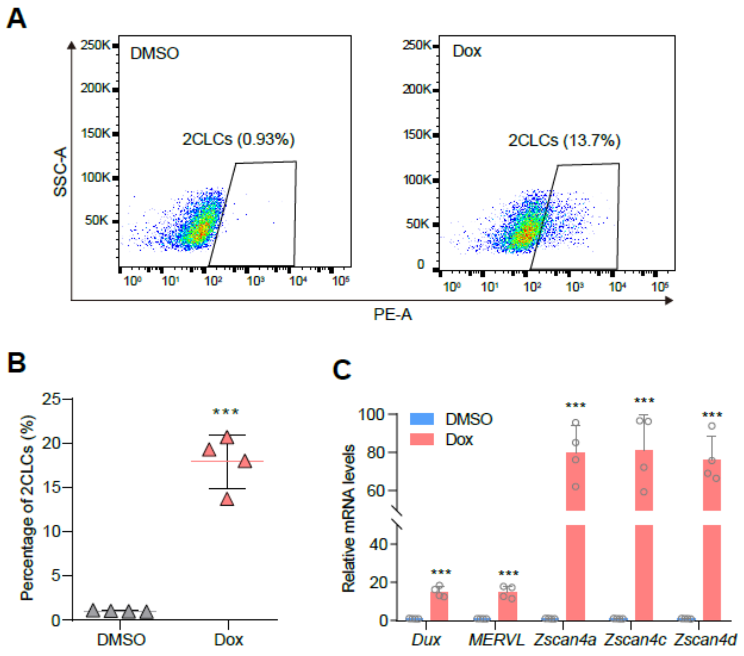

Figure S1. Validation of the inducible 2C-tdTomato system.

(A) Representative flow cytometry showing the percentage of 2C-tdTomato-positive cells in ESCs with (Dox) or without (DMSO) 24 h DUX induction.

(B) The percentage of 2C-tdTomato-positive in ESCs with or without 24 h Dux induction by flow cytometry analysis. Error bars, mean ± SD; ****P* < 0.001, unpaired two tailed *t*-test; *n*= 4.

(C) Detection of 2C gene expression by RT-qPCR in ESCs with or without 24 h DUX induction. Error bars, mean + SD; ****P* < 0.001, unpaired two tailed *t*-test; *n*= 4.

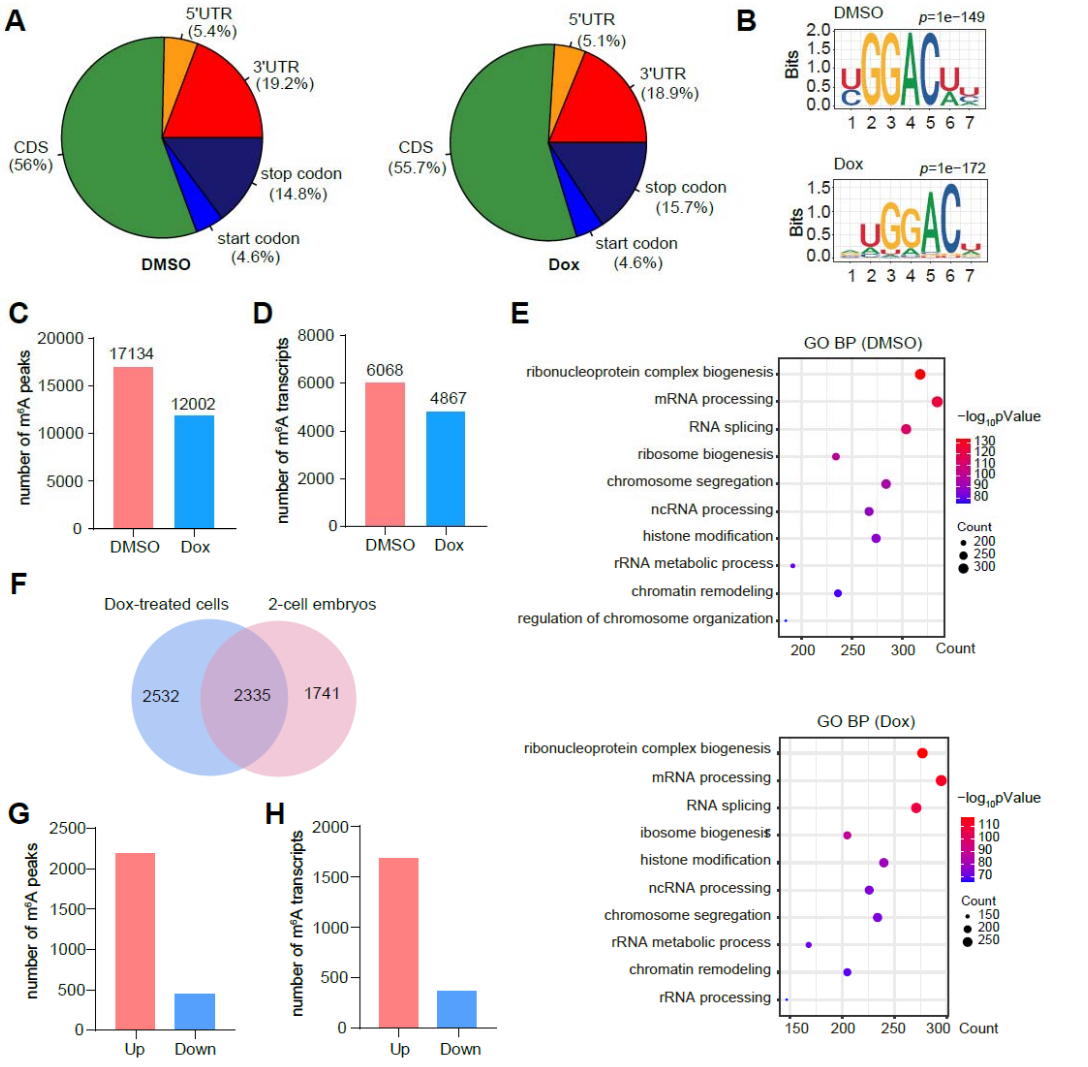

Figure S2. The dynamic m^{6}A methylome during DUX-induced the 2C-like reprogramming.

(A) Pie charts presenting the fraction of m^{6}A peaks within distinct RNA sequence types in ESCs with (Dox) or without (DMSO) DUX induction for 24 h.

(B) Sequence motifs identified within m^{6}A peaks in ESCs with or without 24 h DUX induction. The motif GGAC was highly enriched in m^{6}A peaks.

(C) Number of m^{6}A peaks identified in ESCs with or without DUX induction for 24 h.

(D) Number of m^{6}A-containing transcripts identified in ESCs with or without DUX induction for 24 h.

(E) Bubble plots showing the biological pathways enriched by m^6A-containing transcripts identified in ESCs with (lower panel) or without DUX (upper panel) induction for 24 h.

(F) Venn diagram showing the overlap of m^6A-methylated transcripts in Dox-treated ESCs and 2-cell embryos.

(G) Number of m^6A peaks significantly upregulated (Up) and downregulated (Down) upon DUX induction for 24 h.

(H) Number of m^6A-containing transcripts exhibiting increased (Up) and decreased (Down) m^6A methylation upon DUX induction for 24 h.

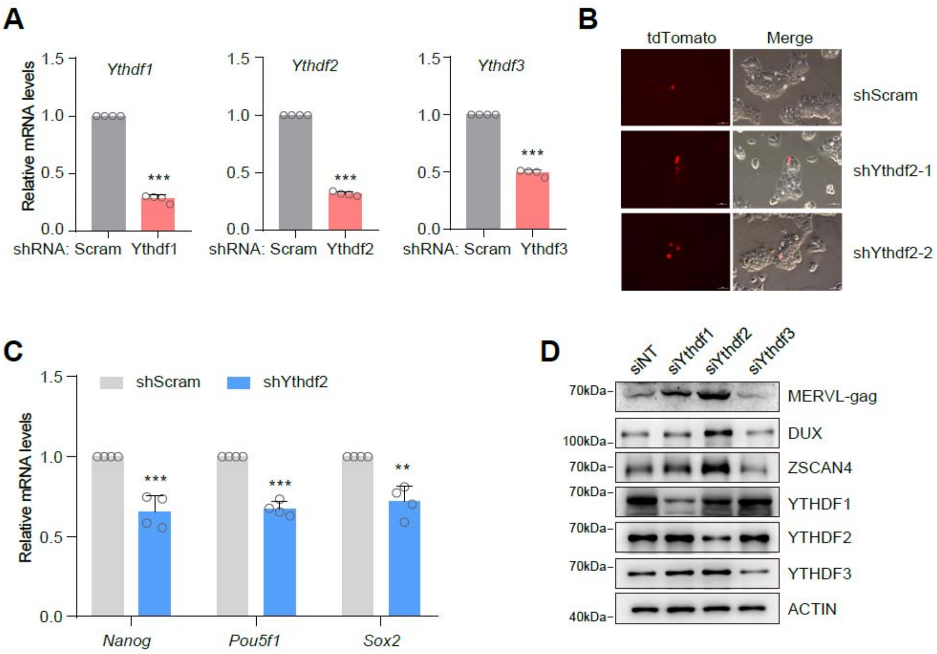

Figure S3. Silencing of YTHDF2 facilitates the 2C-like state in ESCs.

(A) RT-qPCR showing that the knockdown efficiency of *Ythdf1-3* in ESCs. Error bars, mean + SD; ***$P < 0.001$, unpaired two tailed *t*-test; $n = 4$.

(B) Representative image showing 2C-tdTomato-positive cells in control (shScram) and *Ythdf2*-KD (shYthdf2) ESCs. Scale bars, 5 μm.

(C) Detection of pluripotent gene expressions by RT-qPCR in control and *Ythdf2*-KD ESCs. Error bars, mean + SD; **$P < 0.01$, ***$P < 0.001$, unpaired two tailed *t*-test; $n = 4$.

(D) Western blotting showing the expression of YTHDF and 2C-related proteins in cells transfected with siRNAs targeting Ythdf or non-targeting siRNA (NT) as a control.

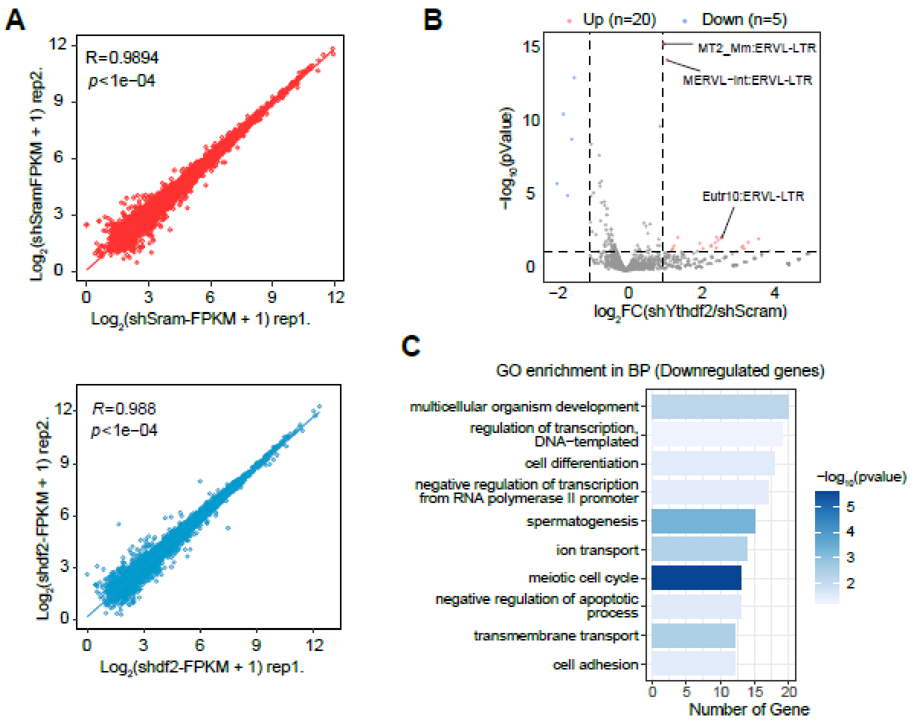

Figure S4. The global transcriptomic changes mediated by YTHDF2.

(A) Scatter plots showing the correlation of gene expression expressed as log2(FPKM + 1) from biological replicates of control and (shScram, upper panel) and *Ythdf2*-KD (shdf2, lower panel) ESCs.

(B) Volcano plots showing changes in expression of transposable elements in response to YTHDF2 depletion.

(C) GO analysis of the biological pathways enriched by downregulated genes upon YTHDF2 depletion.

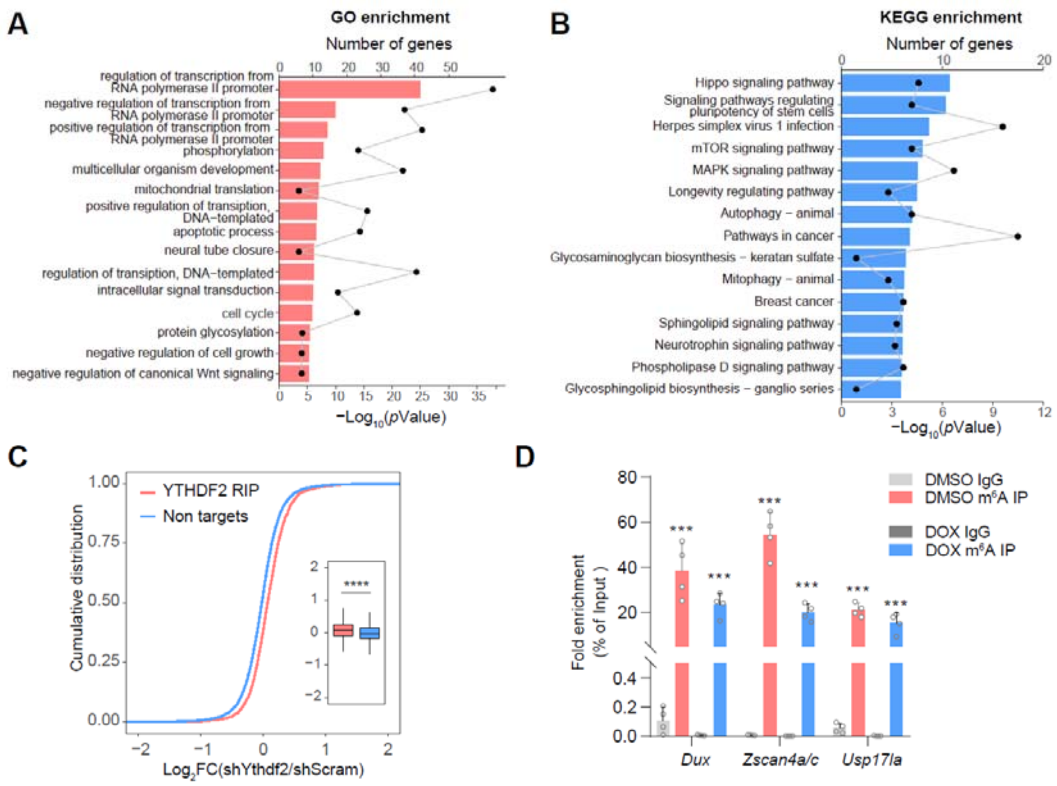

Figure S5. Identification of YTHDF2 target transcripts.

(A) GO analysis of the biological pathways enriched by YTHDF2 binding transcripts.

(B) GO analysis of the KEGG pathways enriched by YTHDF2 binding transcripts.

(C) Cumulative distribution of changes in transcript levels for YTHDF2 target mRNAs (YTHDF2 RIP) and non-target mRNAs (Non targets) between shScramble and shYthdf2.

(D) Detection of m^{6}A levels on *Dux*, *Zscan4a*, *Zscan4c* and *Usp17la* mRNAs in ESCs with (Dox) or without (DMSO) 24 h DUX induction using m^{6}A-RIP-qPCR. Error bars, mean + SD; ***p <0.001, unpaired two tailed t-test; n= 4 independent experiments.

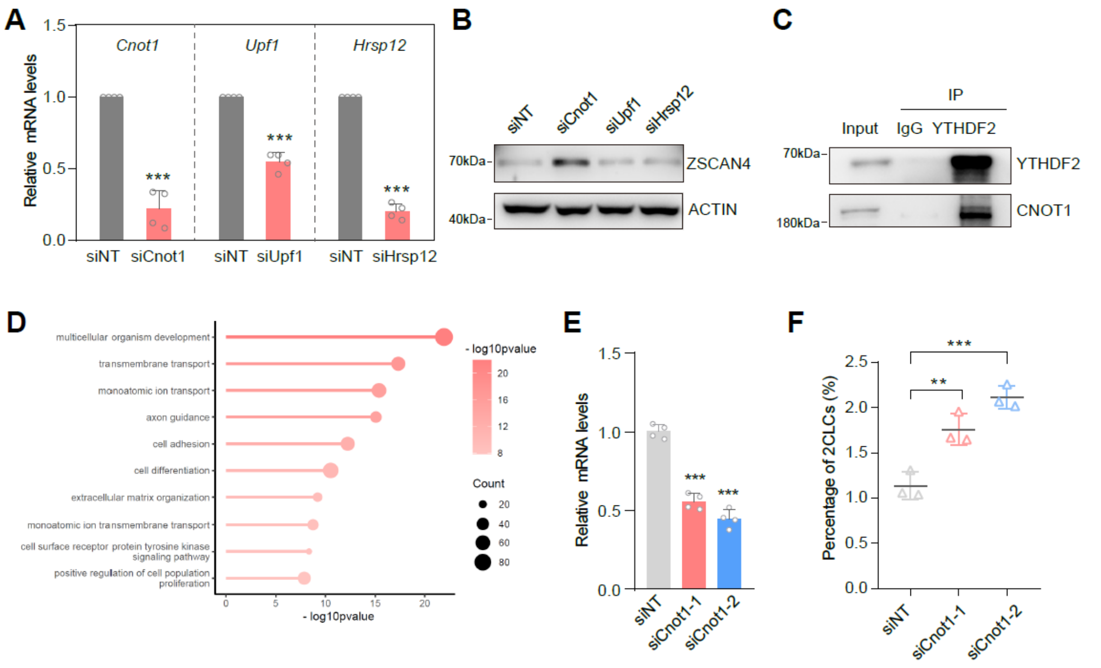

Figure S6. CNOT1 modulates 2C program in ESCs.

(A) RT-qPCR showing that the knockdown efficiency of *Cnot1*, *Upf1* and *Hrsp12* in ESCs. Error bars, mean + SD; ***p <0.001, unpaired two tailed t-test; n= 4 independent experiments.

(B) Western blotting showing the protein expression of ZSCAN4 in cells transfected with siRNA targeting *Cnot1*, *Upf1*, *Hrsp12* or non-targeting siRNA (NT) as a control.

(C) Cell lysates were immunoprecipitated with anti-YTHDF2 followed by immunoblotting using antibody against CNOT1.

(D) GO analysis of the biological pathways enriched by upregulated genes upon YTHDF2 depletion.

(E) RT-qPCR showing that the knockdown efficiency of *Cnot1* in ESCs. Error bars, mean + SD; ***p <0.001, unpaired two tailed t-test; n= 4 independent experiments.

(F) Quantification of the percentage of 2C-tdTomato-positive cells in ESCs transfected with siRNA targeting *Cnot1* or non-targeting siRNA as a control from three biologically independent experiments. Error bars, mean ± SD; **P < 0.01, ***P < 0.001, unpaired two tailed t-test; n= 4.

Table S1. Primers for RT-qPCR used in this study.

Gene name	Forward primer (5'-3')	Reverse primer (5'-3')
Ythdf1	GCCCACAGCTATAACCCTAAA	GTGGATGTCGTCCTCAGAATAG
Ythdf2	CAGTTTGCCTCCAGCTACTATT	GCAATGCCATTCTTGGTCTTC
Ythdf3	GGGAAAGGCCCACTCTATTT	GAGACCAGACACCAGCATAAG
Dux	AAAGGAAGAGCATGTGCCAGC	GCAGTAAGCTGTCCTGGGAAC
Zscan4a	CCTGCCATTCCCTCTGACAT	ATTATTCCACTTTGAACGTCGTT
Zscan4c	TCTTTCTGGTTGGCAGCTTT	GCCAGGCTTCTGTCAAGAAC
Zscan4d	AAGAGGTGAGGTGGAGGAGT	AAGGTCTTTTGCTGGTGCCT
Usp17la	TGGTGGTTGCTCTTTCCTTC	GCTGTTGCCTGTGTTCTGGA
MERVL	ATCTCCTGGCACCTGGTATG	AGAAGAAGGCATTTGCCAGA
Cnot1	CCGACTTGTCTCAGGTGTGG	TCTCGTTCCCTTTTTATTGT
Upf1	TAAAGGATGAGACAGGCGAG	GTCAATGAGGATGGAACGGA
Hrsp12	TACCATCGTTTCTTCTTCTG	GAGCCTGTTTAGCTTCTTCT
ß-Actin	TTGCTGACAGGATGCAGAAG	ACTCCTGCTTGCTGATCCACAT
